# Supplementary figures and images for: The Mla pathway promotes Vibrio cholerae re-expansion from stationary phase
Source: mBio. 2024 Dec 23;16(2):e03433-24. doi: 10.1128/mbio.03433-24 (PMC11796348; doi:10.1128/mbio.03433-24)

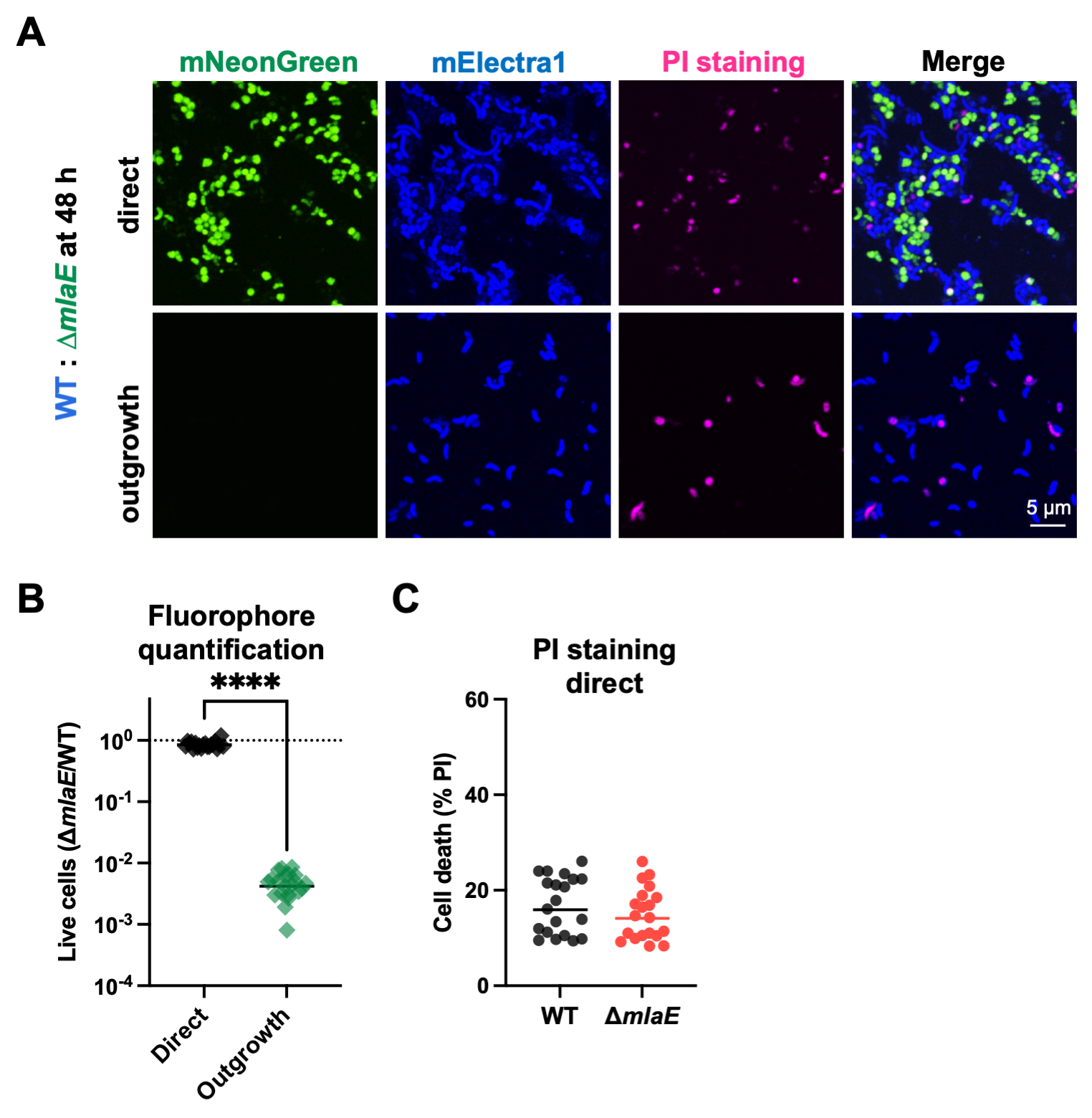

Supplement: Figure S1 — mlaE modifies V. cholerae culturability in stationary phase. [file mbio.03433-24-s0001.tiff]

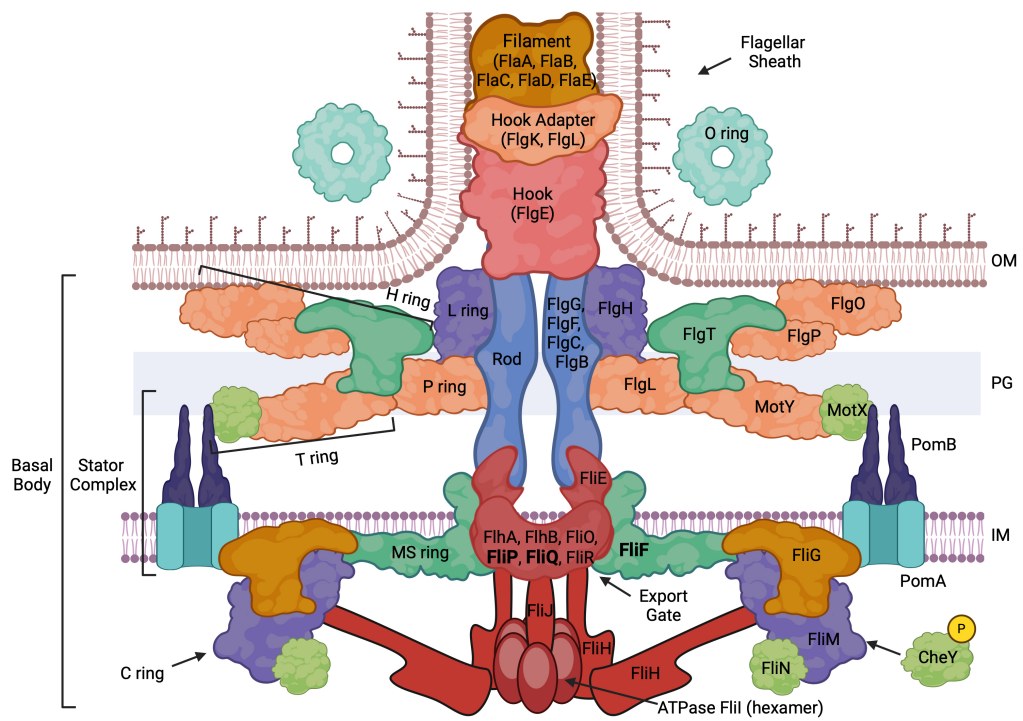

Supplement: Figure S2 — Schematic representation of the V. cholerae flagellum. [file mbio.03433-24-s0002.pdf]

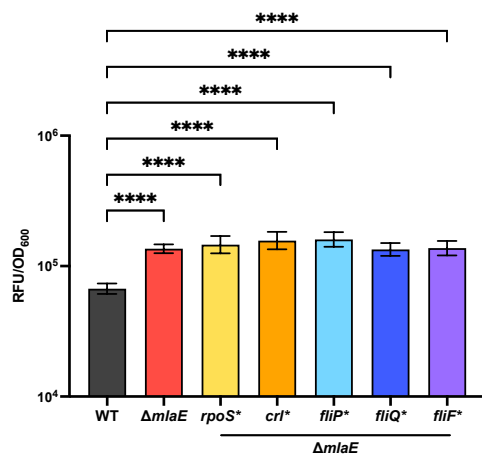

Supplement: Figure S3 — mlaE mutants lose significantly more phospholipids than WT. [file mbio.03433-24-s0003.pdf]
