# Supplementary material for: The Mla pathway promotes Vibrio cholerae re-expansion from stationary phase
Source: mBio. 2024 Dec 23;16(2):e03433-24. doi: 10.1128/mbio.03433-24 (PMC11796348; doi:10.1128/mbio.03433-24)
Supplement: Supplemental legends — Legends for Fig. S1 to S3. [file mbio.03433-24-s0004.docx]

**Fig. S1: *mlaE* modifies *V. cholerae* culturability in stationary phase.** A) Representative images using co-cultures of mElectra1-labeled WT (blue) and mNeoGreen-labeled *mlaE* (green) at 48h; PI (pink) was used to detect dead cells. B) Quantification of fluorophore-positive WT and *mlaE* cells at 48h (n=20 fields; ****P < 0.0001 (Mann-Whitney test). C) Quantification of dead (PI-positive) WT and *mlaE* cells at 48h (n=20 fields). For all graphs, lines represent geometric means and significant differences between data sets are marked by asterisks.

**Fig. S2: Schematic representation of the *V. cholerae* flagellum.** The identified three *mlaE* flagellar suppressor mutations (*mlaE fliP**, *mlaE fliQ** and *mlaE fliF**) are highlighted in bold. Adapted from (1).

1) Lloyd, C.J., and Klose, K.E. (2023). The Vibrio Polar Flagellum: Structure and Regulation. Adv Exp Med Biol *1404*, 77-97. 10.1007/978-3-031-22997-8_5.

**Fig. S3: *mlaE* mutants lose significantly more phospholipids than WT.** Outer membrane vesicle production was measured by incorporation of the fluorescent lipid probe FM4-64 in supernatants of the WT (black), *mlaE* (red)*,* *mlaE rpoS** (yellow), *mlaE crl** (orange), *mlaE fliP** (light blue), *mlaE fliQ** (dark blue) and *mlaE fliF** (purple); the Y-axis shows fluorescence normalized to OD_600_ for each strain. Lines represent geometric means and error bars represent standard deviations of 6 experiments. Significant differences between the data sets are marked by asterisks (****P < 0.0001; Ordinary one-way ANOVA, Multiple comparisons).
